# Psychosis research in Asia: advantage from low prevalence of cannabis use

**DOI:** 10.1038/s41537-016-0002-4

**Published:** 2016-12-13

**Authors:** Tae Young Lee, Jun Soo Kwon

**Affiliations:** Department of Psychiatry, Seoul National University College of Medicine, Seoul, Korea

## First episode psychosis and clinical high risk for psychosis

Since Emil Kraepelin described distinctive clinical course of psychosis, efforts have been globally made to identify aetiology and pathophysiology of psychosis. Earliest studies that conceptualised psychosis as a brain disorder focused on investigating the schizophrenic brain by post-mortem studies and using earlier neuroimaging modalities, such as computed tomography. Significant advances in this field of studies were made in the late twentieth century, in the advent of electrophysiological, magnetic resonance imaging, and functional brain imaging technologies.^1^

Though these technologies were successful in revealing brain abnormalities in psychosis, several confounders, such as the use of psychotropics and chronic institutionalisation, present in chronic psychosis patients arguably obscured the disease effect. With the beginning of twenty-first century, attention was consequently drawn to first-episode psychosis (FEP) patients, who remained unaffected by potential confounds. Attention then shifted to drug-naive FEP patients, when studies found that antipsychotics use shrinks brain volume in psychosis.^2^ These patients, who were free from medication effects, had once been considered clinically unsuitable for research due to florid psychotic symptoms.

Over the last few decades, the concept of clinical high risk (CHR) for psychosis (‘at-risk mental state’ (ARMS), ‘prodromal’, and ‘ultra-high risk’ (UHR) state) has evolved to apprehend pre-psychotic phase. Individuals identified as CHR are at highly increased risk for psychosis and have around 30% of risk for developing the illness within 3 years following the initial presentation.^3^ Their relative distance from overt psychotic symptoms and confounders, such as medication effect, has drawn significant attention of the research community to studying these CHR subjects.

## Cannabis use and CHR research in Asia

Findings that delta-9-tetrahydrocannabinol (THD), which is the principal active ingredient of cannabis, can induce the experience of acute transient psychotic episodes have fostered a considerable growth of literature on the association of cannabis and psychosis. Of particular importance, studies have found that cannabis use increases risk of developing psychosis not only in the general population, but also in CHR subjects,^4^ in whom use was also emphasised as an important predictor for developing psychosis. Accordingly, issues with confounding effect from cannabis and other substance use have emerged in studying psychosis.

Particularly low prevalence of cannabis use is generally reported in Southern and Eastern Asia. Annual cannabis use rates reported by Asian countries actively conducting researches on CHR, such as Hong Kong, Japan, Korea, Singapore, Taiwan, are known to be <0.5%, compared to over 10% has been found in certain American and European countries.^5^ Reasons for markedly low prevalence would be attributable to cultural aspects and strict legislation of illicit substances relevant to these countries. That most of the confounding effect of cannabis is already controlled for, gives a notable advantage to Asian countries in studying psychosis.

The negligible cannabis and other substance-use rates in Asian countries might be able to, at least in part, explain why, for instance, Klauser *et al*.^6^ have reported no abnormality of brain volume or cortical thickness in CHR subjects. However, contrary results also exist, as well.^7^

Large-sample, multi-centre collaborative work has been a rising trend in psychosis research, especially in regards to CHR subjects. The rise of consortiums and joint projects, such as NAPLS, PRONIA, and PSYSCAN, has shown that this collaborative effort allow for larger samples and more robust power. In this context, large Asian cohorts and consortium would be able to realise the unique advantages provided for psychosis research in Asia, and thereby offer insights into the current disparities in research.
